# Technical tip - Using peroperative distraction in calcaneal fracture surgery after correction of hindfoot varus

**DOI:** 10.1016/j.tcr.2026.101386

**Published:** 2026-05-22

**Authors:** E.R.J. Bruns, J.A. Halm, M. Swords, A.K. Sands, T. Schepers

**Affiliations:** a Trauma Unit, Amsterdam UMC, Amsterdam Movement Sciences, Meibergdreef 9, 1105 AZ, Amsterdam, the Netherlands; b Michigan Orthopedic Center, University of Michigan Health-Sparrow, 1215 Michigan Ave, Lansing, MI, USA; c New York-Presbyterian Lower Manhattan Hospital, New York, NY, 10038, USA

**Keywords:** Calcaneus, Fracture, Minimal invasive, Percutaneous, Mini-distractor

## Abstract

**Introduction:**

Calcaneus fracture surgery requires a well thought-out but pragmatic approach. The general fracture dislocation pattern involves shortening and varus deformation. The sinus tarsi approach (STA), which is performed in the lateral decubitus position, has become a more popular option due to its decreased soft tissue complication rate, while retaining the ability to perform anatomic reduction and fixation of most calcaneus fractures. As the STA technique requires indirect reduction, an assistive devise can be used to facilitate the reduction.

**Technique:**

The STA is performed in the lateral decubitus position by performing the following five consecutive steps: first, the medial joint surface (sustentaculum tali) is reduced and temporarily stabilized by inserting a medial plantar 1.6 mm K-wire. Second, the 5.0 mm Schanz pin is inserted perpendicularly into the infero-lateral tuberosity, and the calcaneus tuber length and varus are corrected while restoring medial wall congruency. The previously placed two 1.6 mm k-wires are advanced from the plantar medial tuber into the sustentaculum fragment. Third, the mini distractor is mounted after inserting 3.0 mm Schanz pins into the base of the neck of the talus, making sure the tip engages the far cortex. Fourth, distraction is applied using the mini-distractor to further restore height and length of the tuber and body of the calcaneus and to clear space in the posterior facet which aides in the reduction of the lateral joint fragment. Lastly, internal fixation is applied by means of a posterior facet/tuber extension type of lateral anatomical plate and independent angled axial screws.

**Conclusion:**

This current technical tip shows how to correct varus first and then uses the same Schanz pin for the use of a mini-distractor on the lateral side of the hindfoot.

The technique described places the patient in a lateral decubitus position without the need for medial access to the hindfoot. Reduction and fixation of intra-articular calcaneal fractures safely can be performed with limited lateral incisions.

## Highlights

• Describes a lateral mini-distractor technique for displaced intra-articular calcaneus fractures
• Anatomic reduction is enabled via sinus tarsi approach without medial exposure in lateral position.
• Employs a single 5.0 mm Schanz pin for sequential varus correction and distraction
• Minimally invasive method reduces soft-tissue trauma and operative duration reducing infection rates.

## Introduction

There are many ways human beings have found to injure themselves and sustain fractures. The resulting fractures may be described in a limited number of conceptual patterns. Calcaneal fractures are often the cause of a fall from height or motor vehicle accidents, are no exception to having a wide number of variations. The calcaneus is inferior to the talus, which acts as a wedge against the top of the calcaneus, resulting in the calcaneus receiving much of the force of an axial load. Secondly, the calcaneus is a thin-walled bone filled with soft cancellous bone and a large empty space, best described in the origin of its name from Calx-Calcis - ‘limestone’ in Latin - a loan from the Greek χάλιξ, which indicates a pebble or gravel. The transmitted energy causes a fracture with loss of height, incongruence of the subtalar joint, widening, and a varus malignment [1]. Optimal functional results in recovery require the treating specialist to properly correct these deformities.

Traditionally, fractures are known to result in shortening and displacement of the original anatomy of the affected bone. Traction is one of the oldest treatment techniques used to correct deformity. However, due to its anatomical shape and location, calcaneal fractures result in complex deformities. Therefore, traction itself cannot lead to a satisfactory reduction of the articular face [2]. In order to reconstruct the calcaneus surgery was found to be helpful. In the past 20 years many surgeons have switched from the older extensile approach to using a combination open reduction of the subtalar joint via the STA and percutaneous reduction and fixation of the overall anatomy.

**Fig. 1:**
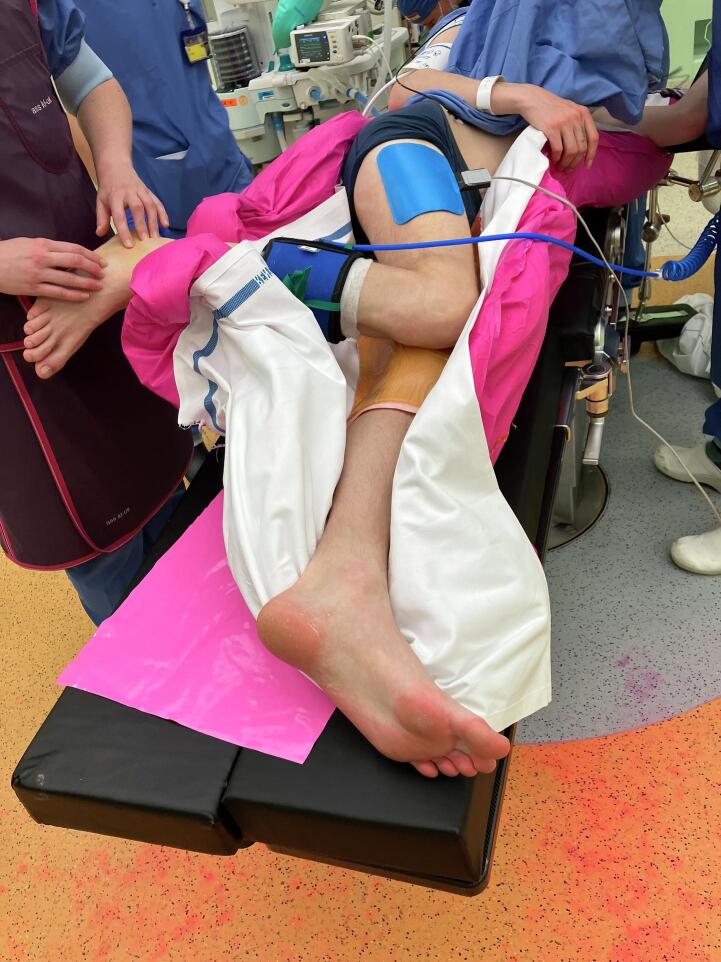
Lateral decubitus position.

The use of temporary per-operative distraction in displaced intra-articular fractures of the calcaneus is not a new concept and has been described in various forms. Grala et al. described their technique in 2009 with the use of the large femoral distractor whilst performing an extended lateral approach (ELA) [3]. The benefit of this technique is that a large force can be applied. The downside is that the size of the distractor makes the fine tuning of the subtalar reduction unwieldly. Yu et al. used a smaller device but placed it on the medial side of the calcaneus after inserting two 2.5 mm K-wires from lateral to medial through the calcaneal tuberosity and talus [4]. Wireman et al. also used a distraction device on the medial side, with one 2.0 mm pin placed in the tuberosity and the other into the sustentaculum [5]. Placement of distraction devices on the medial side might in fact be the best position to counteract the varus deformity caused by the injury. However, it could be considered more difficult to place the device on the medial side, with the patient in a lateral decubitus position (Fig. 1) which is preferable both for a sinus tarsi approach and the extended lateral approach.

To strive towards functional minimalism in the treatment of calcaneal fractures, we describe our preferred technique when using the small distraction/compression device positioned on the lateral side, while using the same Schanz pin used for varus correction.

## Technique

**Fig. 2:**
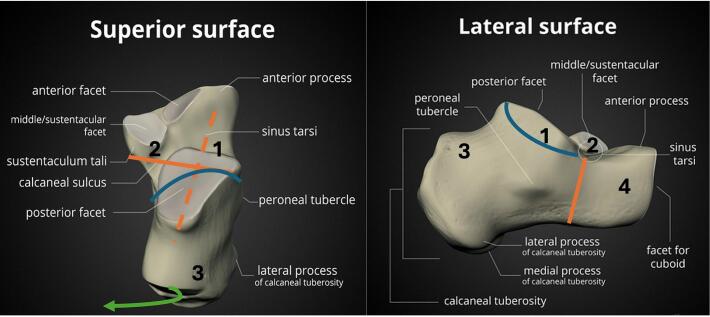
Pathoanatomy of calcaneal fractures.

The calcaneus mostly fractures in a predictable way based on its anatomy and the position of the talus with respect to the calcaneus. The application of an axial force causes the calcaneus to breaks into four large pieces (more if there is increased amount of energy which increases the amount of both displacement and comminution) as shown in Fig. 2 [1]. As a result of the lateral offset of the calcaneal axis in relation to the axis of the talus, the calcaneal tuber displaces into varus as well as laterally.

The common fragments are the lateral subtalar joint fragment (1), the medial subtalar joint fragment (2), also called the sustentaculum fragment (or constant fragment), the tuberosity fragment (3), the anterior process fracture (4) (sometimes split in a lateral and medial part).

**Fig. 3:**
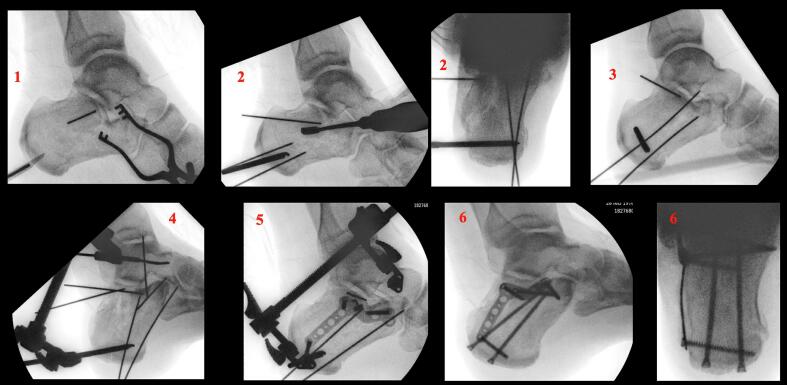
Reduction and application of mini-distractor.

**Fig. 4:**
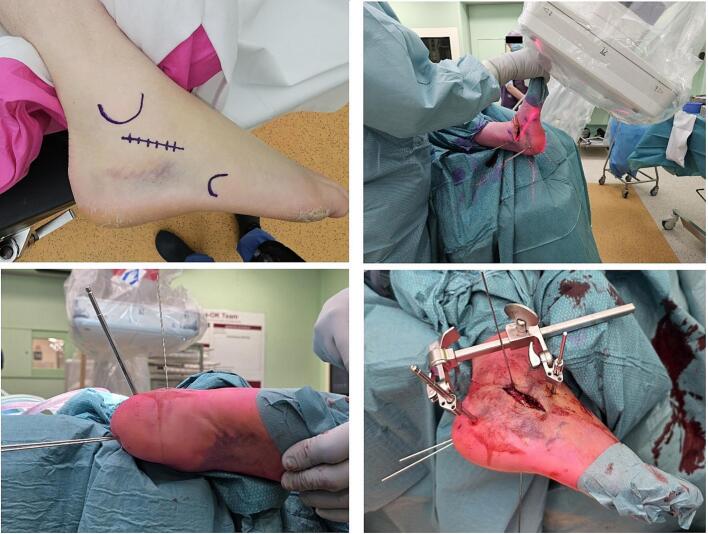
Clinical imaging perioperatively.

The steps of surgical reconstruction of the calcaneus depend on the Essex-Lopresti fracture pattern (joint depression or Tongue type). The following steps are visualized peroperatively in by means of imaging in Fig. 3 and clinically in Fig. 4. After performing the STA, the canalis tarsi is debrided by removing Hoke's fat, hematoma and ligaments between talus and calcaneus. The lateral wall is exposed raising a full thickness flap starting at the sinus tarsi incision using a no.15 blade. Care is taken to not injure the peroneal tendons or sural nerve with the large periostal elevator required for this step. The inferior peroneal retinaculum needs to be elevated off the lateral wall to ensure the plate is not placed into the interosseous space under the lateral wall blow out.

The medial sustentaculum (constant) fragment is reduced against the talus and temporarily fixed to the talus with a 1.6 mm K-wire from medial - plantar (Fig. 3-1).

**Fig. 5:**
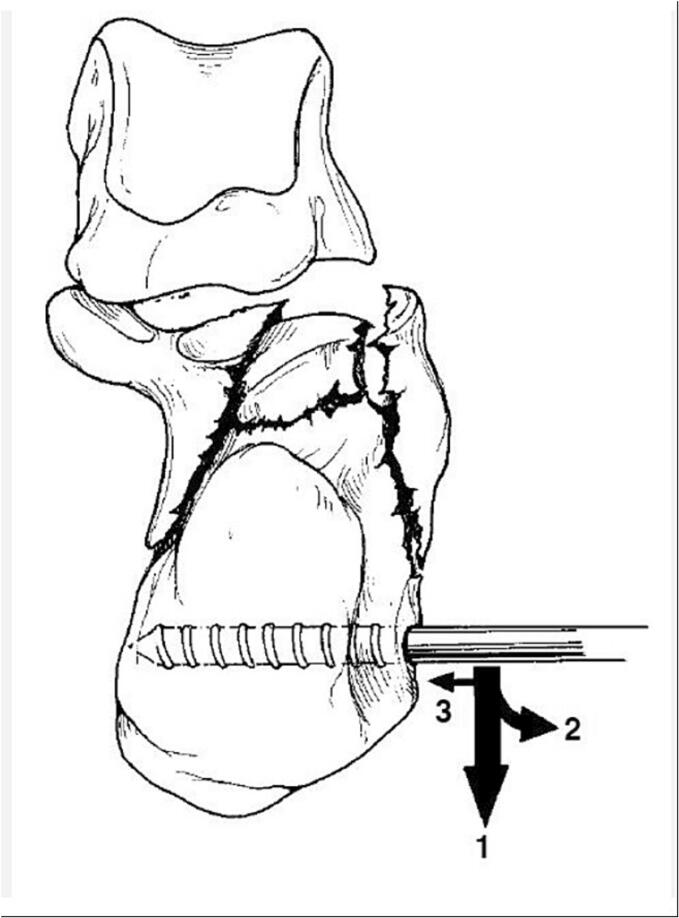
5.0 Schanz pin in order to restore axis, height and alignment.

**Fig. 6:**
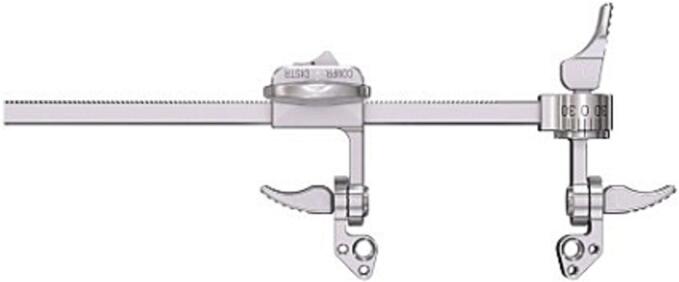
Mini distraction/compression device.

Subsequently, a 5.0 mm Schanz pin is inserted into the distal part of the tuber, perpendicular to the calcaneal axis. By first creating more varus and heaving the tuber over the medial spike of the sustentaculum initial reduction is achieved. (Fig. 5) Next, the tuber is placed in a neutral position and pushed further medial (Fig. 3-2). Subsequently, two 1.6 mm K-wires that were previously inserted partially into the inferior tuber, stopping just short of the level of the fracture, are advanced across the reduced fracture and into the sustentaculum fragment (Fig. 3-3). The mini-distractor (Fig. 6) is installed after inserting an additional 3.0 mm Schanz pin in the neck of the talus, or rarely in the case of ipsilateral talus fracture, in the tip of the distal fibula. By means of distraction, space is created in the subtalar joint to reduce the lateral joint fragment (Fig. 3-5). If necessary, the medial sustentaculum K-wire may be removed from the talus allowing the surgeon to tip the joint for better visualization of the entire joint surface. The use of a headlamp or a small joint arthroscope may also aid in reduction. After reduction (and temporary k-wire placement) is complete and radiographs (lateral, axial and Brodén) confirm the reduction, internal fixation is applied consisting of a lateral anatomical plate and axial screws (Fig. 3-6).

In tongue type injuries, the medial sustentaculum (constant) fragment is reduced against the talus and temporarily fixed to the talus with a 1.6 mm K-wire placed from medial - plantar (Fig. 3-1). In some cases, this step is omitted resulting in a so called joint first approach.

Subsequently, an axial 5.0 mm Schanz pin is inserted in the superior tongue fragment to restore the calcaneus length, height, and axis (Fig. 5). Next, the two 1.6 mm K-wires that were previously inserted partially into the tuber are advanced into the fragment (Fig. 3-3). The mini-distractor is installed (Fig. 6) after inserting an additional 2.5/3.0 mm Schanz pin in the neck of the talus or the tip of the distal fibula and the tuber. By means of distraction, space is created in the subtalar joint to optimize articular reduction (Fig. 3-4, 5). If necessary, the medial sustentaculum K-wire is may be removed allow better visualization of the joint surface. After reduction is complete and radiographs (lateral, axial and Brodén) confirm the reduction, internal fixation is applied consisting of a more postero-lateral anatomical plate and more vertically oriented screws (which must ideally engage the plantar tuber cortex) are placed (Fig. 3-6).

**Fig. 7:**
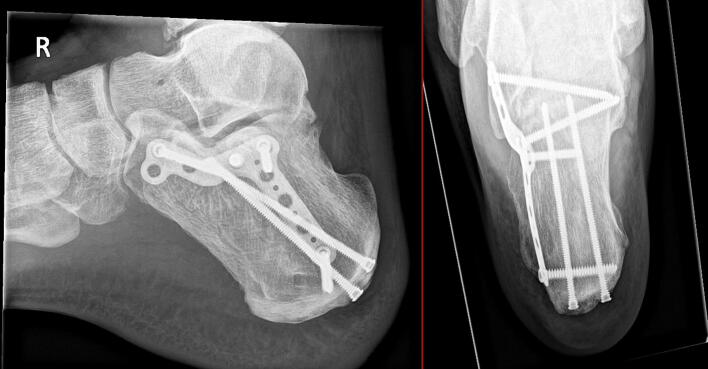
Clinical imaging 1 year postoperatively.

Patient recovered without any complications. The American Orthopedic Foot and Ankle Society (AOFAS) Score was 83 after one year: pain 30/40, function 43/50 (3 points decrease in activity, 1 decrease in distance, 3 points in subtalar movement) alignment 10/10. Clinical imaging 1 year postoperatively is shown in Fig. 7.

## Discussion

This paper describes the practice of applying traction for reduction of calcaneus fractures on the lateral side in a safe and functional way, and as such enhances the perioperative approach.

Fundamentally, placing the distractor laterally challenges the concept that only the medial side is suited to place a distraction device in order to counteract varus deformity [6]. The anatomy of the calcaneus results in fracture patterns that require a combination of reduction forces to be applied in which traction is only a segment and thus, as described above, the varus deformity could also be treated separately by means of a manually manipulable Schanz pin perpendicular to the lateral wall. This pin can also be replaced by a Kirschner, Steinmann-Codvilla, or Denham (centrally threaded) pin [7].

The STA and concomitant reduction in operative duration compared to the extensile lateral approach, is preferable to keep infection rates as low as possible [3]. This approach is less stressful on the vulnerable soft tissue envelope surrounding the calcaneus. The concept of ‘backwards reasoning’ used by renown figures such as Sherlock Holmes (originating from the mind of Sir Arthur Conan Doyle, a physician), applied to calcanear fractures defines the steps in its treatment to restore height, congruence, width and correction of varus, in a reversed order as the typical patho-anatomy that originates form the injury mechanism. Since the deformation always is a cumulative result, applying a distractor laterally is easier and contributes to anatomical reposition in combination use with the other reduction techniques described above.

In conclusion, calcaneal fractures demand a well thought-out and pragmatic approach. In a lateral decubitus position used for a sinus tarsi approach, application of a mini distractor on the lateral side of the foot combined with a Schanz pin to correct varus, is a suitable alternative to medial distraction without compromising anatomical reduction or plate placement.

## CRediT authorship contribution statement

**E.R.J. Bruns:** Writing – review & editing, Writing – original draft, Conceptualization. **J.A. Halm:** Writing – review & editing, Writing – original draft, Supervision. **M. Swords:** Writing – review & editing. **A. Sands:** Writing – review & editing, Supervision. **T. Schepers:** Writing – review & editing, Writing – original draft, Supervision, Conceptualization.

## Funding

This research did not receive any specific grant from funding agencies in the public, commercial, or not-for-profit sectors.

## Declaration of competing interest

The authors declare that they have no known competing financial interests or personal relationships that could have appeared to influence the work reported in this paper and did not receive any specific grant from funding agencies in the public, commercial, or not-for-profit sectors.
